# Supplementary material for: Self-assessed health status and obesity vulnerability in rural Louisiana: A cross-sectional analysis
Source: PLoS One. 2023 Jun 16;18(6):e0287181. doi: 10.1371/journal.pone.0287181 (PMC10275442; doi:10.1371/journal.pone.0287181)
Supplement: S1 File — (DOCX) [file pone.0287181.s002.docx]

(D2D) Tensas CDC 1809 Community Survey

Q1
Welcome to the Tensas Healthy Communities Survey! Please enter the SURVEY ID of the house being surveyed below.

Q2 Enter SURVEY ID from address list:

________________________________________________________________

Q3 Zip Code

________________________________________________________________

Q4 **Has the participant been read the informed consent and given consent to participate in this survey?**

- Yes, they would like to begin the survey.
- No, they decline to be surveyed (Do not administer survey)

Q5 Are you at least 18 years of age?

- Yes
- No (Do not administer the survey)

Q6 Have you lived in Tensas Parish for at least 6 months?

- Yes
- No

Q7 Do you do ***at least some*** of the food shopping for this household?

- Yes
- No

Q8 How many children under the age of 18 are in your household?

- 0
- 1
- 2
- 3
- 4
- 5
- 6
- 7 or more children
- Choose not to answer

Q9 How many adults (18 and over) are in your household (including yourself)?

- 1
- 2
- 3
- 4
- 5
- 6
- 7 or more adults
- Choose not to answer

Q10 Read each statement carefully and then indicate how strongly you agree or disagree with each statement.

Q11 Please tell us how strongly you agree or disagree with the following statements.

|  | Strongly disagree | Disagree | Agree | Strongly agree | Choose not to answer |
| --- | --- | --- | --- | --- | --- |
| I am satisfied with the selection of fresh fruits and vegetables in Tensas. |  |  |  |  |  |
| Since the corona virus outbreak, there have been more resources available in Tensas to help me feed my family. |  |  |  |  |  |
| Since the corona virus outbreak, I have purchased more food in Tensas than I usually do. |  |  |  |  |  |
| My household lost regular income because of the corona virus outbreak. |  |  |  |  |  |

Q12 What is the name of the store where you buy **most**of your food? (Choose one.)

- Mac’s Fresh Market (skip to Q15)
- Grocery or supermarket outside of Tensas Parish (ex: Walmart, Winn Dixie)
- Dollar store in Tensas Parish (skip to Q15)
- Dollar store outside of Tensas Parish
- Convenience or corner store in Tensas Parish (skip to Q15)
- Convenience or corner store outside of Tensas
- None of these stores (skip to Q14)
- Choose not to answer (skip to Q14)

Q13 What would you say is the **main** reason you buy most of your food at this store?  Is it because of... (Choose one.)

- Price
- Location
- Quality of products
- Variety of products
- Your friends/relatives shop there
- Customer service
- Store quality
- Other (describe below) ________________________________________________
- Choose not to answer

Q14 What is the name of the store that you buy the most food from **in Tensas Parish**? (Choose one.)

- Mac’s Fresh Market
- A Dollar Store in Tensas
- A Convenience or Corner Store in Tensas
- I do not purchase food in Tensas (skip to Q27)
- Choose not to answer (skip to Q27)

Q15 What would you say is the **main** reason you buy food at this store in Tensas?  Is it because of... (Choose one.)

- Price
- Location
- Quality of products
- Variety of products
- Your friends/relatives shop there
- Customer service
- Store quality
- Other (describe below) ________________________________________________
- Choose not to answer

Q16 How often do you usually shop for food at this store? (Choose one.)

- More than once a week
- Once a week
- Once every 1-2 weeks
- Once a month
- Other (describe): ________________________________________________
- Choose not to answer

Q17 How do you usually travel to this store? (Select all that apply.)

- Walk
- Bicycle
- Council on Aging bus
- Drive your own car
- Get a ride (for free)
- Pay for a ride
- Other (describe): ________________________________________________
- Choose not to answer

Q18 About how much of your food usually comes from this store in Tensas? (Choose one.)

- None
- A little
- Some
- Most
- All
- Choose not to answer

Q19 How would you rate the price of fresh fruits and vegetables at this store? Would you say the price is... (Choose one.)

- Very inexpensive
- Not expensive
- Somewhat expensive
- Very expensive
- This store does not sell fresh fruits and vegetables.
- Choose not to answer

Q20 For the following questions, please tell us how strongly you agree or disagree with the following statements **about the store you stated you buy the most food from in Tensas**.  As a reminder, questions about unhealthy foods mean those foods often considered to be high in sugar, salt, fat and calories, such as candy, chips, soda, desserts, and so on.

|  | Strongly Disagree | Disagree | Agree | Strongly Agree | Choose not to answer |
| --- | --- | --- | --- | --- | --- |
| At this store, I notice signs that encourage me to purchase healthy foods. |  |  |  |  |  |
| At this store, I often buy food items that are located near the cash register. |  |  |  |  |  |
| At this store, I notice a lot of signs and displays encouraging me to buy unhealthy foods. |  |  |  |  |  |
| I feel safe from crime traveling to and from this store. |  |  |  |  |  |
| I feel safe from crime when shopping at this store. |  |  |  |  |  |

Q27 Please select **ALL** of the places where you purchase fresh fruits and vegetables from? **(Select ALL that apply.)**

- Mac’s Fresh Market
- Corner store or convenience store in Tensas
- Grocery or supermarket outside of Tensas (Ex: Wal-Mart, Brookshire’s)
- Fruit and/or vegetable truck or roadside stand **in** Tensas
- Fruit and/or vegetable truck or roadside stand **outside** of Tensas
- Farmer's market **in** Tensas Parish
- Farmer’s market **outside** Tensas Parish
- Other (describe): _____________________________________________
- I do not purchase fresh fruits or vegetables.
- Choose not to answer

Q28 Does anyone in your household receive food from a food pantry?

- Yes
- No (Skip to Q36)
- Choose not to answer (Skip to Q36)

Q29 What location does your household gets **the most food from**? (Choose one.)

- Oneonta Baptist Church food pantry (on Hubbard St. in St. Joseph)
- Shepard Center (on Plank Rd. in St. Joseph)
- Tensas Council on Aging meal site (on Plank Rd. in St. Joseph)
- Other Food Pantry (describe): ____________________________________________
- Choose not to answer

Q30 The next set of questions will be about the pantry that you stated your household gets the most food from (from Q29).

Q31 How long would it take you to get there from your home if you **walked** there?

- 10 minutes or less
- 11 to 20 minutes
- 21 to 30 minutes
- More than 30 minutes
- Choose not to answer

Q32 How satisfied are you with **how often** you and others in your household can get food from this location?

- Very dissatisfied
- Somewhat dissatisfied
- Somewhat satisfied
- Very satisfied
- Choose not to answer

Q33 How satisfied are you with **the amount** of food that you and others in your household get at this location?

- Very dissatisfied
- Somewhat dissatisfied
- Somewhat satisfied
- Very satisfied
- Choose not to answer

Q34 How satisfied are you with **the variety** of food that you and others in your household get at this location?

- Very dissatisfied
- Somewhat dissatisfied
- Somewhat satisfied
- Very satisfied
- Choose not to answer

Q35 Which types of foods do you want but do not usually get from this location?

- Fresh fruits and vegetables
- Healthy protein food items such as lean meats and poultry, fish, or shellfish
- Skim or low-fat dairy products, such as milk, yogurt, or cheese
- Whole grain foods
- Other (describe): _______________________________________________
- I get all the types of food I want at this location.
- Choose not to answer

Q36 This next set of questions is about walking and biking. Think about a typical month and think about how often you walk to get from one place to another. This could include, for example, if you drove to town, went to the post office, and then walked to the library.

Q37 In a typical month, how often do you **walk** to get from one place to the following locations?

|  | Never | Once per month | 2-3 times per month | Weekly | 2-4 times per week | Almost every day or daily | Choose not to answer |
| --- | --- | --- | --- | --- | --- | --- | --- |
| School |  |  |  |  |  |  |  |
| Work |  |  |  |  |  |  |  |
| Library |  |  |  |  |  |  |  |
| Church |  |  |  |  |  |  |  |
| Friend or relative's home |  |  |  |  |  |  |  |
| Park |  |  |  |  |  |  |  |
| Grocery Store |  |  |  |  |  |  |  |
| Other location (describe):  _________ |  |  |  |  |  |  |  |
| Other location (describe):  _________ |  |  |  |  |  |  |  |

Q38 Do you have access to a working bicycle?

- Yes
- No (Skip to Q40)
- Choose not to answer (Skip to Q40)

Q39 In a typical month, how often do you bike to get from one place to the following locations?

|  | Never | Once per month | 2-3 times per month | Weekly | 2-4 times per week | Almost every day/daily | Choose not to answer |
| --- | --- | --- | --- | --- | --- | --- | --- |
| School |  |  |  |  |  |  |  |
| Work |  |  |  |  |  |  |  |
| Library |  |  |  |  |  |  |  |
| Church |  |  |  |  |  |  |  |
| Friend or relative's home |  |  |  |  |  |  |  |
| Park |  |  |  |  |  |  |  |
| Grocery store |  |  |  |  |  |  |  |
| Other location (describe):  __________ |  |  |  |  |  |  |  |
| Other location (describe):  __________ |  |  |  |  |  |  |  |

Q40 Does anyone else in your household walk or bike?

- Yes
- No (Skip to Q47)
- Choose not to answer (Skip to Q47)

Q41 Think of the person in your household, other than yourself, **who is most likely to walk or bike** to get from one place to another.  Then, answer the following questions with that household member in mind.

Q42 How old is this household member?

________________________________________________________________

Q43 What is their gender?

________________________________________________________________

Q44 In a typical month, how often does the household member you just described walk to get from one place to the following locations?

|  | Never | Once per month | 2-3 times per month | Weekly | 2-4 times per week | Almost every day/daily | Choose not to answer |
| --- | --- | --- | --- | --- | --- | --- | --- |
| School |  |  |  |  |  |  |  |
| Work |  |  |  |  |  |  |  |
| Library |  |  |  |  |  |  |  |
| Church |  |  |  |  |  |  |  |
| Friend or relative's home |  |  |  |  |  |  |  |
| Park |  |  |  |  |  |  |  |
| Grocery store |  |  |  |  |  |  |  |
| Other location (describe):  __________ |  |  |  |  |  |  |  |
| Other location (describe):  __________ |  |  |  |  |  |  |  |

Q45 Does this household member have access to a working bicycle?

- Yes
- No (Skip to Q47)
- Choose not to answer (Skip to Q47)

Q46 In a typical month, how does this household member bike to get from one place to the following locations?

|  | Never | Once per month | 2-3 times per month | Weekly | 2-4 times per week | Almost every day/daily | Choose not to answer |
| --- | --- | --- | --- | --- | --- | --- | --- |
| School |  |  |  |  |  |  |  |
| Work |  |  |  |  |  |  |  |
| Library |  |  |  |  |  |  |  |
| Church |  |  |  |  |  |  |  |
| Friend or relative's home |  |  |  |  |  |  |  |
| Park |  |  |  |  |  |  |  |
| Grocery store |  |  |  |  |  |  |  |
| Other location (describe):  __________ |  |  |  |  |  |  |  |
| Other location (describe):  __________ |  |  |  |  |  |  |  |

Q47 For the following statements, please tell me how strongly you agree or disagree.

|  | Strongly Disagree | Disagree | Agree | Strongly Agree | Choose not to answer |
| --- | --- | --- | --- | --- | --- |
| I feel safe from crime walking or riding a bike near my home in the **daytime**. |  |  |  |  |  |
| I feel safe from crime walking or riding a bike near my home in the **nighttime**. |  |  |  |  |  |
| Stray dogs keep me from walking or biking near my home. |  |  |  |  |  |
|  | Strongly Disagree | Disagree | Agree | Strongly Agree | Choose not to answer |
| The roads around my home have a place to walk or ride a bike next to the road (shoulder, bike lane, path, etc.). |  |  |  |  |  |
| There are sidewalks on most of the roads in the area around my home. |  |  |  |  |  |
| There are sidewalks in Tensas that connect places so that you can walk from place to place (like connecting a store to the post office). |  |  |  |  |  |
|  | Strongly Disagree | Disagree | Agree | Strongly Agree | Choose not to answer |
| I feel safe from traffic walking or riding a bike near my home. |  |  |  |  |  |
| I admire people who walk or bike for their transportation, rather than driving . |  |  |  |  |  |
| Most people who walk or bike for transportation do so because of their financial situation. |  |  |  |  |  |
| I would be embarrassed to walk or bike to get from one place to another, for my transportation. |  |  |  |  |  |
| Only people that are up to no good walk or bike to get from one place to another for transportation. |  |  |  |  |  |

Q48 In the past week, on how many days have you done a **total of 30 minutes or more of physical activity**, which was enough to make you breathe harder. This may include sports, exercise, and brisk walking or biking for recreation or to get to and from places. (Choose one.)

- 0 days
- 1 day
- 2 days
- 3 days
- 4 days
- 5 days
- 6 days
- 7 days
- Choose not to answer

We have just a few more questions to help us understand more about you and your household. All of your answers will remain private and secure.

Q50 In general, would you say your health is...

- Excellent
- Very good
- Good
- Fair
- Poor
- Choose not to answer

Q51 What is your age?

________________________________________________________________

Q52 What is your gender?

________________________________________________________________

Q53 Are you of Hispanic or Latino origin?

- Yes
- No
- Choose not to answer

Q54 Race (choose all that apply)

- American Indian or Alaskan Native
- Asian
- Black or African American
- Native Hawaiian or Other Pacific Islander
- White
- Choose not to answer

Q55 Current employment status?

- Employed (full or part-time)
- Seasonally employed
- On disability
- Homemaker
- Retired
- Student
- Not currently employed
- Choose not to answer

Q56 If employed, how many hours did you work last week?

________________________________________________________________

Q57 Did your household participate in any of these food programs **prior to the coronavirus pandemic** (in 2019)? (choose all that apply)

- Food Stamps/SNAP
- Women, Infants, and Children Program (WIC)
- WIC Farmer's Market Nutrition Program
- Senior Farmers Market Vouchers
- Commodities Program (CSFP)
- Local Meal Sites (such as those provided by Council on Aging)
- Other (describe): ______________________________________________
- None of these
- Choose not to answer

Q58 Does your household participate in any of these food programs **now**? (choose all that apply)

- Food Stamps/SNAP
- Women, Infants, and Children Program (WIC)
- WIC Farmer's Market Nutrition Program
- Senior Farmers Market Vouchers
- Commodities Program (CSFP)
- Local Meal Sites (such as those provided by Council on Aging)
- Other (describe):_______________________________________________
- None of these
- Choose not to answer

Q59 How often in the past 12 months would you say you were worried or stressed about having enough money to buy nutritious meals?

- Never
- Rarely
- Sometimes
- Usually
- Always
- Choose not to answer

Q60 How far have you gone in school?

- Up to 8th grade
- Some high school, no diploma
- High school graduate, GED
- Some college credit, no degree
- Trade/technical/vocational training
- Associate's degree
- Bachelor's degree
- Master's degree or higher
- Choose not to answer

Q61 At any time in your life, have you ever been treated unfairly because of your race or ethnicity?

- Yes
- No (Skip to Q63)
- Choose not to answer (Skip to Q63)

Q62 When shopping for food in Tensas, how often have any of the following things happened to you because of your race/ethnicity?

|  | Never | Sometimes | About half the time | Most of the time | Always | Choose not to answer |
| --- | --- | --- | --- | --- | --- | --- |
| You are treated with less courtesy or respect than other people. |  |  |  |  |  |  |
| You receive poorer service than other people. |  |  |  |  |  |  |
| People act as if they think you are not smart. |  |  |  |  |  |  |
| People act as if they are afraid of you. |  |  |  |  |  |  |
| You are threatened or harassed. |  |  |  |  |  |  |

Q63 How many working vehicles are there in your household?

________________________________________________________________

Q64 How many working bicycles are there in your household?

________________________________________________________________

**Congratulations! You have completed the Healthy Communities Survey.  There's only one thing left to do--receive your FREE Swag Bag!**

To return this survey and receive your Swag Bag:

Mail to:

Healthy Communities

202B Knapp Hall

Baton Rouge, LA 70808

OR

Drop off at the Tensas Extension Office:

4589 LA-605

St. Joseph, LA 71366
