# Supplementary material for: Self-assessed health status and obesity vulnerability in rural Louisiana: A cross-sectional analysis
Source: PLoS One. 2023 Jun 16;18(6):e0287181. doi: 10.1371/journal.pone.0287181 (PMC10275442; doi:10.1371/journal.pone.0287181)
Supplement: Attachment — Submitted filename: Response to reviewers.docx [file pone.0287181.s003.docx]

**RESPONSE TO REVIEWERS**

Journal Requirements:

When submitting your revision, we need you to address these additional requirements.

1. Please ensure that your manuscript meets PLOS ONE's style requirements, including those for file naming. The PLOS ONE style templates can be found at <https://journals.plos.org/plosone/s/file?id=wjVg/PLOSOne_formatting_sample_main_body.pdf> and <https://journals.plos.org/plosone/s/file?id=ba62/PLOSOne_formatting_sample_title_authors_affiliations.pdf>.

**Response:** The revised version is structured as per PLOS ONE’s style requirements.

2. In the ethics statement in the Methods, you have specified that verbal consent was obtained. Please provide additional details regarding how this consent was documented and witnessed, and state whether this was approved by the IRB.

**Response:** After obtaining the verbal consent, we document the consent in written form. The consent procedure was approved by the LSU Agricultural Center IRB# HE 20-24.

3. Please include your ethics statement in the Methods section of your manuscript. In the Methods section of your revised manuscript, please include the full name of the institutional review board or ethics committee that approved the protocol, the approval or permit number that was issued, and the date that approval was granted.

**Response:** The research procedure follows the ethical standards of the IRB. The LSU Agricultural Center Institutional Review Board (IRB) approved this study (IRB# HE20-24) on May 15, 2020

4. In your Data Availability statement, you have not specified where the minimal data set underlying the results described in your manuscript can be found. PLOS defines a study's minimal data set as the underlying data used to reach the conclusions drawn in the manuscript and any additional data required to replicate the reported study findings in their entirety. All PLOS journals require that the minimal data set be made fully available. For more information about our data policy, please see <http://journals.plos.org/plosone/s/data-availability>.

Upon re-submitting your revised manuscript, please upload your study’s minimal underlying data set as either Supporting Information files or to a stable, public repository and include the relevant URLs, DOIs, or accession numbers within your revised cover letter. For a list of acceptable repositories, please see <http://journals.plos.org/plosone/s/data-availability#loc-recommended-repositories>. Any potentially identifying patient information must be fully anonymized.

Important: If there are ethical or legal restrictions to sharing your data publicly, please explain these restrictions in detail. Please see our guidelines for more information on what we consider unacceptable restrictions to publicly sharing data: http://journals.plos.org/plosone/s/data-availability#loc-unacceptable-data-access-restrictions. Note that it is not acceptable for the authors to be the sole named individuals responsible for ensuring data access.

**Response:** We do not have restrictions regarding data sharing. We can provide anonymized data set underlying the results described in the manuscript as a supplementary information file.

Additional Editor Comments:

Unfortunately, in the current format the article cannot be considered for publication. The introduction of the study is confusing, in the methods the statistical analyses need to be detailed, and the presentation of the results and discussion is very confusing. Also, the conclusion needs to be revised. The reviewers made several suggestions and I believe that after an extensive revision the study can be considered for publication again.

**Response:** We are grateful to the editor for providing us with very constructive suggestions and feedback that helped significantly improve our manuscript. We address all comments and suggestions in the current version. Basically, we have done the following:

- Slightly modified the title to more reflect the research question and study settings.
- Restructured the introduction to enhance clarity and ensure content flow.
- Added more details about the methods adopted and statistical analyses employed in the study.
- Separated results and discussion section.
- Revised conclusions to make it more relevant with policy implications.

Reviewer #1: I congratulate the authors for the work developed. During the reading of the paper I had some doubts that are listed below.

**Response:** We thank the reviewer for recognizing the importance of our work and for providing constructive feedback, which we address below.

- I suggest that the authors leave the sections of the article more delimited, in some moments there are questions about methodology presented in the results and vice-versa.

**Response:** We have rewritten the Introduction section and expanded the data and methods section to enhance clarity. We believe that the updated version is more delimited and clearer.

- Introduction: It is not entirely clear what is the gap in the literature that the study will answer. Once the data presented in the introduction demonstrates that the factors that influence obesity are already well described in the literature. I suggest structuring the introduction more directly and making the justification for the work clearer.

**Response:** We have restructured the introduction section and added justifications for the work explicitly in the updated version as (page 4, lines 83-102):

*The existing studies on SAH have centered their analyses on how and to what extent gender disparity (Berggren and Ljunge, 2021), healthcare access (Courtemanche et al., 2020), digital divide (Tavares, 2020), financial strain (Mazeikaite et al., 2019), and fuel poverty (Llorca et al., 2020) influence SAH. However, studies that focus on rural residents regarding the determinants of SAH are only a few. Furthermore, despite obesity prevalence rapidly increasing in the rural areas, to our knowledge, there is not any study that explores vulnerability level of households or individuals concerning obesity. The index-based measure to assess the vulnerability level is widely used and provides a picturesque of underlying exposure to risks and level of preventive efforts of households or communities (Cutter et al., 2003; Balica et al., 2012). Such index-based measure also provides objective criterion that aids planning and implementation of obesity-related interventions. Thus, we attempt to fill this gap in the literature by investigating two major research questions: i) What factors influence SAH status in rural areas? ii) What is the vulnerability level of the rural residents in terms of obesity? To this end, we employ ordinal logit regression model and principal component analysis on data from community surveys in three rural counties in Louisiana, USA. The results show that grocery store choice and exercise frequency significantly influence SAH status, besides sociodemographic variables such as gender, education, and interaction between age and race. Furthermore, our findings show that around two-third of the respondents are vulnerable to obesity, thus necessitating prompt preventative actions to promote healthy wellbeing in rural Louisiana.*

- What criteria were used to select the three rural communities included in the study?

**Response:** In 2018, the Louisiana State University (LSU) AgCenter entered into a cooperative agreement with the Centers for Disease Control and Prevention (CDC) to address obesity in six rural Louisiana counties that had obesity prevalence rates over 40%. To inform and evaluate their efforts, the Louisiana State University (LSU) AgCenter collected representative data in three of the six grant counties using the RELFA survey. The three counties of interest were selected based on the research team’s perspective that these communities were best situated to evaluate measurable change as a result of grant efforts. We have added text in the updated version about the selection of three rural communities as (line 128-130, page 6):

*These three counties were selected because obesity prevalence rate is over 40% and they remain priority areas of the Centers for Disease Control and Prevention (CDC) to address obesity.*

- What were the inclusion and exclusion criteria for the participants? It would be important to make clear what criteria were adopted in conducting the study.

**Response:** We have included the inclusion and exclusion criteria for the participants in the updated version as (page 7, line 157-162):

*Participants were eligible to participate in the survey if they lived in the county of interest, were at least 18 years of age, and did some of the food shopping in the household. Survey refusals were not included in the replacement scheme. Written informed consent was obtained, and surveys were either collected via iPad using the offline Qualtrics application or on paper.*

- What software was used to perform the statistical analyses?

**Response:** We used Stata 17 to perform statistical analyses in the paper. We mention that in the updated version as (page 14, line 260):

*All statistical analyses were conducted using Stata 17.*

- In the results it appears that the chi-square test was performed, but this type of information is not available in the methodology. It would be important to make clear in the methodology all the statistical analyses that were performed.

**Response:** We did not perform separate chi-square test for regular hypothesis testing in our manuscript. The chi-square value that appears in the logistic regression model is the value of test statistic for goodness of fit tests for logistic regression. Since, it is a usual convention not to mention the details about the diagnostic tests of the model, we did not include any information about the chi-square test in the methodology section. However, we have provided details about the methodology adopted to analyze factors affecting self-assessed health and obesity vulnerability index in detail in the present version.

- The discussion needs to be reformulated, because the authors focused too much on presenting that the results agree with previously developed studies. It would be important to provide more robust explanations for the results found.

**Response:** We have separated the results and discussion section in the present version. More detailed explanations are added to justify the results as (page 20-23, line 360-432):

*The findings from the study imply that grocery store choice and exercise frequency have significant associations with the overall SAH status, besides demographic factors such as gender, education, and age-race interaction. This is not unusual because local grocery stores provide few food choices compared to big box grocery stores. The negative influence of local grocery store choice implies that lower-income families have limited access to supermarkets and other healthy food retail outlets that provide varieties of affordable and nutritious foods. These limitations with local stores may be associated with poorer dietary choices and consequently reporting poor SAH. Similarly, rural infrastructure does not well support walking, running, or biking activities, thus exercise frequency is likely to be hampered that have implications for the SAH status of rural residents.*

*The significant value of race by age interaction term suggests that racial disparity in SAH is affected by the age of the respondent. In other words, with every additional year of age, Black respondents are less likely to value their SAH to be in the better categories. An unexpected result is a significantly positive association between race and SAH. A possible explanation is that more than 80% of respondents are black or African American, and almost 74% of these respondents reported their health status as good or excellent, which is largely reflected in the SAH. The positive association of having children with better SAH is in line with the findings by Fritzell and Gähler (2017).*

*The marginal effects for age had a significant and positive impact on the ‘fair’ and ‘good’ levels of health status but a negative sign on the ‘excellent’ level. In general, older people may have more disabilities and compromised health conditions that lead to poor SAH ratings compared with younger adults. The results are consistent with previous studies, including Andersen et al. (2007), McFadden et al. (2008), and Jurewicz and Kaleta (2020).*

*Generally, regular physical activity is one of the most important things people can do to improve their health. The more often an individual is physically active, the better their health status. The results are in line with previously published studies that suggested a positive relationship between physical activity and SAH (Abu-Omar et al., 2004; Galán et al., 2010). The significantly positive marginal effect of higher educational attainment on the excellent SAH category may be because educational attainment is associated with better health (Franks et al., 2003; Tavares, 2020). Regarding the marginal effects of race, our results are consistent with the findings by Lee et al. (2007) and Krok-Schoen et al. (2021). The authors found that older African Americans were more likely to rate SAH as poor. The Centers for Disease Control and Prevention (2008) also reported that Black adults are more likely to report their general health status as fair or poor compared to White adults.*

*The results relating to OVI show that more than 65% of the respondents fall into vulnerable segments, either in the moderate, high, or very high category. Such a high level of vulnerability is an indication of the severity of obesity-related risks in the study area, thus prompting policy actions to cope with obesity in the study area. Furthermore, the OVI estimates suggest that the respondents with SAH as good, very good, and excellent are less vulnerable to obesity than those reporting fair or poor. This implies that respondents' self-assessment of their health status is correct. The vulnerability to obesity decreased with the increasing frequency of exercise. The rate of decrease of OVI with 30-minute exercise is steeper up to 4 days per week, after which it starts to increase. This U-shaped relationship between exercise and OVI is interesting but unclear, thus needs further investigation. Having more vehicles in the home is also associated with higher OVI. One unusual observation from our study is that OVI is increasing when the number of bikes in a household is >3. This needs additional investigation and might imply that using bikes is more important than merely having them. Having sidewalks on roads and the perception that traffic is safe for walking or biking around the residential area is associated with lower OVI. This has implications for Louisiana, where sidewalks are less common, and the crime rate is higher than in other states in the US. The results further imply that there is wide heterogeneity in the distribution of OVI by different categories of variables. Thus, targeting programs could be more useful in combating the obesity-related problem.*

*Policymakers and health practitioners need credible evidence before making decisions to address obesity in rural communities. This study uses representative data to determine risk and protective factors for obesity that could inform evidence-based interventions for decision-makers. First, results indicating that low-income households are more at risk for obesity reinforce the need for evidence-based nutrition and physical activity interventions targeted at low-income rural residents. Second, results show a lower risk of obesity associated with the presence of sidewalks and perceptions of safe walking and biking support policies and funding that improve pedestrian infrastructure and rural road design to encourage active transportation in rural communities. Lastly, the associations between grocery store choice and obesity vulnerability support policy and interventions that encourage improved access to healthy foods, both in terms of availability and affordability, in the rural food retail sector.*

*Although our study sheds light on how different factors influence SAH and the underlying vulnerability to obesity among rural residents, there are a few caveats with our analyses. First, only three Louisiana counties were included, which might limit the generalization of the study; however, these counties are still the priority of the Centers for Disease Control (CDC) programs for preventing obesity. Second, we consider socioeconomic and health-related factors in constructing OVI; however, further research using more comprehensive data about actual diet choices could strengthen our findings. Third, survey respondents may not always have been the primary food purchaser of the household which could have impacted responses to questions about food shopping behaviors and experiences. Lastly, the inferences drawn using cross-sectional data could be bolstered by using household-level longitudinal data.*

Reviewer #2:

I appreciate the opportunity to evaluate the article in question. This is an article that sought to investigate the influence of sociodemographic factors on self-rated health and determine vulnerability to obesity in rural communities. This is an important theme for public health and a special one for working with a population group outside the urban context.

**Response**: We are very thankful for the reviewer for being appreciative of our work and also providing insightful comments and suggestions that our review has considered below.

I point out some necessary changes in the article:

SUMMARY - the objective needs to be direct. I suggest deleting the first sentence of the objective. The methods need to bring detail beyond PCA analysis. The conclusions presented are final considerations. I suggest a rewrite.

**Response:** We have reformatted the objective. Additional details regarding the data collection and empirical strategy has been included. The methods section is expanded to bring detail beyond PCA analysis. Similarly, conclusions have been rewritten.

INTRODUCTION: It is too long. I think it deserves a restructuring. The research problem is reduced in the introduction and should be more detailed. Until line 61, the introduction could be reduced to a single paragraph. Abstract and Introduction Objectives are different, I suggest standardization.

**Response:** We have restructured the introduction section along with more detailed discussion to the research problem. Abstract and Introduction Objectives have been rewritten.

METHODS: I believe that the initial part of the methods is missing. It is not presented with the methodological design of the study - I believe it is a cross-sectional study. This needs to be informed. I suggest writing it according to STrengthening the Reporting of OBservational studies in Epidemiology - STROBE.

**Response:** This is a cross-sectional study and we clarify this in the ‘Survey design and data collection’ section as (page 5, line 125-128):

*This research uses representative cross-sectional data from the Rural Eastern Louisiana Food Accessibility and Active Transportation (RELFA) survey to investigate predictors of SAH and determine obesity vulnerability in three rural Louisiana counties: East Carroll, Saint Helena, and Tensas (Fig 1).*

Regarding PCA, I suggest explaining the process of analysis better. Was it measured eigenvalues > 1.0, defined according to the scree plot for the extraction of the components? This is not clear. Why was the coefficient and not the OR presented?

**Response:** It is a usual convention to use eigenvalues >1 according to the scree plot for the extraction of components, especially during factor analysis with multiple components. However, as the first component explains most of the variation in the data, we are interested in extracting only the first component as a weight for the variables under consideration while constructing an index. Using only the first component as a weight is a common practice in the literature. Therefore, we did not have to resort to scree plot for the extraction of the components. However, we add explanations about the PCA for clarity in the updated version as (page 13-14, line 236-260):

*Using standardized values, we run* *principal component analysis (PCA) to calculate the weight of each indicator used to construct the OVI. PCA is a popular non-parametric tool that allows the use of a wide range of indicators while also providing credible unequal weighting to the indicators (Maison et al., 2001; Jolliffe and Cadima, 2016).*

*Before running the PCA, the Kaiser-Meyer-Olkin (KMO) test was conducted to examine the sampling adequacy of data. The KMO measure was 0.593, indicating that the data are satisfactory for PCA analysis. The index value was obtained by multiplying the component 1 score from PCA (Table 2) with the standardized value of each variable and summing them. The weights obtained from the PCA vary between -1 to +1 and the magnitude of the weights is an indication of the relative contribution of indicators to the OVI. We used the integrated vulnerability assessment framework of IPCC (2014) with the REFLA survey data for calculating the OVI. The vulnerability of a respondent to obesity is defined as the net value obtained by subtracting the level of preventive efforts from the overall sensitivity level:*

$Vulnerability=Sensitivity -Preventive efforts$	*(5)*

*Based on this definition, the OVI can be expressed as*

| $OVI =\sum_{k=1}^{9} x_{ik}w_{x_{k}} -\sum_{p=1}^{7} y_{ip}w_{y_{p}}$*, x є X, y є Y, and i = 1, …, N.* | *(6)* |
| --- | --- |

*where OVI denotes the obesity vulnerability index, and x and y refer to the sensitivity and preventive efforts indicators, respectively. w stands for the weights obtained as first component loadings from principal component analysis for the k^th^ or p^th^ indicator, and i indexes respondents. Assigning weights based on the first component values is widely practiced and reliably assigns weights to construct an empirically valid index (Cutter et al., 2003; Filmer and Pritchett, 2001). A higher value of OVI indicates a higher vulnerability to obesity and vice versa; however, this is not an absolute but rather a subjective measure to facilitate comparative ranking among sampled respondents. The resulting OVI provides insights for strategic planning and weighing alternatives for coping with obesity.*

We replace the coefficients with the Odds Ratio in the Table 3 for ease of interpretation. We retained marginal effects in the table because it is more interpretable than the odds ratio and helps determine how a marginal change in one explanatory variable changes the distribution of the response variable.

**Table 3.** Estimated odds ratio from the ordered logit model

| Variables | *Dependent variable = Self-assessed health (SAH)* |
| --- | --- |
|  | Odds ratio |
| *Employment* | 1.833  (0.700) |
| *Children* | 1.771**  (0.408) |
| *Race (African American=1)* | 6.998**  (5.441) |
| *Age* | 0.996  (0.011) |
| *Gender (Female=1)* | 0.693**  (0.108) |
| *Exercise frequency* | 1.596***  (0.268) |
| *SNAP* | 0.819  (0.144) |
| *Education* |  |
| High school | 1.282  (0.289) |
| Some college or training | 2.107***  (0.548) |
| Associate degree or above | 2.056***  (0.568) |
| *Store choice* |  |
| Jong’s Super | 0.527**  (0.155) |
| Greensburg Market | 0.388***  (0.120) |
| Mac’s Fresh Market | 0.641  (0.187) |
| Walmart-Winn Dixie | 0.657  (0.195) |
| *Race*$\times$*Age* | 0.967***  (0.012) |
| *Race*$\times$*Employment* | 0.806  (0.327) |
| *Employment*$\times$*Children* | 0.665  (0.205) |
| Log-likelihood | -671.14 |
| LR $\chi^{2}$ (17) | 135.77*** |
| Pseudo R^2^ | 0.092 |
| *p*-value | 0.000 |
| Number of observations | 682 |

Notes: Standard errors in parentheses. ***<0.01, **<0.05, and *<0.10.

RESULTS AND DISCUSSION: I think there was a mistake in the writing. I suggest separating results from discussion. I suggest presenting the PCA analysis in the results separately.

**Response:** We have separately presented the PCA analysis in the results section. Moreover, the results and discussion sections are also separated.

The tables need to be redone - they present the code of the variables, but I believe that this is not necessary for this article. Presenting the discussion this way ended up compromising the depth of the discussion, becoming superficial and not evolving the results found.

**Response:** The table 1 has been redone by replacing the code of the variables with the actual name.

The study does not present the limitations, I suggest its inclusion.

**Response:** We acknowledge the limitations of the study in the present version as:

*Although our study sheds light on how different factors influence SAH and the underlying vulnerability to obesity among rural residents, there are a few caveats with our analyses. First, only three Louisiana counties were included, which might limit the generalization of the study; however, these counties are still the priority of the Centers for Disease Control (CDC) programs for preventing obesity. Second, we consider socioeconomic and health-related factors in constructing OVI; however, further research using more comprehensive data about actual diet choices could strengthen our findings.* *Third, survey respondents may not always have been the primary food purchaser of the household which could have impacted responses to questions about food shopping behaviors and experiences. Lastly, the inferences drawn using cross-sectional data could be bolstered by using household-level longitudinal data.*

CONCLUSION: The way it is presented it is not a conclusion of the study but final considerations. I suggest rewriting the conclusions.

**Response:** We have rewritten the conclusion section as (page 24, line 435-454):

*Obesity is a rapidly emerging challenge for public health practitioners in the rural US. Thus, the information about the correlates of SAH status and the underlying vulnerability of the communities to obesity is critical to addressing burgeoning obesity-related problems. In this study, we used representative survey data from rural Louisiana to examine the socio-economic factors influencing SAH and calculated the OVI for low-income communities. The results suggest that interventions to increase grocery store choice and exercise infrastructure could promote SAH, thus lowering vulnerability to obesity. Since, majority (~65%) of the residents fall in the obesity-vulnerable segment, rolling out targeted prevention and control measures is very necessary to minimize the rural-urban gap in obesity prevalence.*

*Our findings further show that there is a wide heterogeneity in the vulnerability level of the rural residents, thus necessitating diversity in intervention groups instead of a blanket approach while implementing obesity prevention and control measures. The findings of this research provided insights into a policy discussion about designing an effective and efficient suite of interventions in low-income communities to fight obesity. As currently structured, only a few low-income communities are included in this study with an exclusive focus on northern Louisiana. Future studies could include all low-income communities to evaluate how individuals’ SAH level changes along with their socio-economic characteristics at a regional or state level. OVI estimates could be further strengthened by a more extensive study incorporating a broader range of variables at both household and community levels. All these topics are left for future research.*
